# Homozygous *HOXC13* Variant Causes Pure Hair and Nail Ectodermal Dysplasia via Reduction in Protein Stability

**DOI:** 10.1155/2024/6420246

**Published:** 2024-07-01

**Authors:** Virginia Clowes, Xiaolun Ma, Hannah Maude, Catherine Dennis, Qing Gao, Geraldine Quinn, Edel A. O'Toole, Kapila Batta, Inês Cebola, Wei Cui

**Affiliations:** ^1^ North West Thames Regional Genetics Service Northwick Park and St Mark's Hospitals, London HA1 3UJ, UK; ^2^ Section of Genetics and Genomics Department of Metabolism Digestion and Reproduction Imperial College London, London W12 0NN, UK; ^3^ Institute of Reproductive and Developmental Biology Department of Metabolism Digestion and Reproduction Imperial College London, London W12 0NN, UK; ^4^ Department of Dermatology Luton and Dunstable NHS TRUST, Luton LU1 2LJ, UK; ^5^ Centre for Cell Biology and Cutaneous Research Blizard Institute Faculty of Medicine and Dentistry Queen Mary University of London, London E1 2AT, UK; ^6^ Department of Dermatology West Hertfordshire Hospitals NHS Trust, Vicarage Road, Watford WD18 0HB, UK; ^7^ Origins of Child Health and Disease Centre for Paediatrics and Child Health (PaeCH) Faculty of Medicine Imperial College London, London W12 0NN, UK

**Keywords:** clinical genetics, ectodermal dysplasia, *HOXC13*, PHNED, protein stability

## Abstract

Pure hair and nail ectodermal dysplasia (PHNED) is a congenital disorder characterized by reduced or absent hair and dystrophic nails. PHNED is caused by pathogenic variants in genes involved in hair and nail development, including *HOXC13*. Previously reported biallelic *HOXC13* pathogenic variants led to PHNED by either disrupting protein expression through nonsense-mediated decay or altering the DNA-binding affinity of the homeobox domain of HOXC13. Here, we report a case of *HOXC13*-related PHNED with a rare homozygous variant, c.931C>T, p.Arg311Trp. Similarly to previously reported missense variants, p.Arg311Trp resides in the homeobox domain of HOXC13 and was assumed to lead to the decreased transcriptional activity of target genes. However, in contrast with previously reported variants, *in vitro* overexpression assays revealed that the p.Arg311Trp variant decreases HOXC13 protein stability, which is corroborated by a series of *in silico* predictions. Computational models further suggest that p.Arg311Trp results in a structural rearrangement with loss of interhelical connection between Arg311 in *α*-helix 3 and Glu276 in *α*-helix 1. Altogether, our results suggest a novel molecular mechanism causative of PHNED, whereby biallelic pathogenic variants in *HOXC13* may result in decreased protein stability and consequently decreased transcriptional activity of target genes essential for hair and nail development.

## 1. Introduction

Pure hair and nail ectodermal dysplasia (PHNED) is a congenital disorder characterized by reduced or absent hair and dystrophic nails, without abnormalities of other ectodermal or nonectodermal structures [1]. Whilst various clinical forms of PHNED have been described, only three causative genes, *KRT85*, *KRT74,* and *HOXC13*, have been identified thus far. Both *KRT85* and *KRT74* encode keratins, which are major structural components of hair and nails [2, 3]. Pathogenic biallelic variants in *KRT85* and *KRT74* cause autosomal recessive PHNED type 4 (OMIM 602032) and type 7 (OMIM 614929), respectively. Biallelic variants in *HOXC13* are associated with autosomal recessive PHNED type 9 (OMIM 614931) and have been reported in individuals of multiple ethnicities, although the condition is very rare, reported in only 8 families worldwide so far [4].

In *HOXC13*-associated PHNED, the phenotype ranges from mild hair loss and sparse hair to complete atrichia (involving the scalp, eyes and eyebrows, body, and secondary sexual hair). The nail phenotype ranges from fragile nails and koilonychia to severe nail dystrophy, with all 20 nails usually involved. Examination of scalp skin from an affected individual demonstrated a reduced number of hair follicles, with disorganised hair shafts lacking the normal layered structure [5]. Lacrimal duct obstruction has been reported in one *HOXC13*-related PHNED family and is a feature of other ectodermal dysplasias [6].

*HOXC13* is a member of the highly conserved *HOX* gene family that is essential for embryonic patterning and encodes, HOXC13, a transcription factor that is mainly expressed in hair follicles and other skin appendages. HOXC13 is an important regulator of hair and nail growth and development, through transcriptional regulation of downstream target genes, including genes encoding keratins, keratin-associated proteins, and other transcription factors [7–11]. In a skin sample from an individual with PHNED type 9, HOXC13 target genes showed significantly decreased or absent mRNA expression [5]. HOXC13-null mice manifested a lack of external hair, abnormal nails, and vertebral anomalies; however, skeletal anomalies have not been reported as part of the human phenotype [12]. Here, we report the 9^th^ family affected with *HOXC13*-related PHNED caused by a rare homozygous variant, c.931C >T, p.Arg311Trp, in the *HOXC13* gene.

## 2. Material and Methods

### 2.1. Participants and Genetic Analysis

Trio whole genome sequencing with gene panel analysis was undertaken through the 100,000 Genomes Project. Algorithms incorporating variant frequency, familial inheritance, variant impact, and genotype-phenotype association were used. The prioritised variant list then underwent manual review and validation by the local laboratory and clinical team. Variant interpretation followed the American College of Medical Genetics (ACMG) [13] and UK Association for Clinical Genomic Science (ACGS) [14] guidelines.

### 2.2. Variant Pathogenicity Analyses

Results for variant pathogenicity predictors were obtained either from the tool's website (listed in the Web Resources section), the Ensembl Variant Effect Predictor (VEP) (release 109) [15], or the VarSome browser [16]. AlphaMissense [17], which utilises a deep learning model trained on population frequency and predicted protein structure, was queried using the HOXC13 UniProt ID (P31276) and the p.Arg311Trp variant. PolyPhen-2, which predicts pathogenicity based on features that capture protein structure and function [18], was run with the WT HOXC13 protein sequence (UniProt P31276) and the p.Arg311Trp variant as input. VarSome was used to obtain results from SIFT (sorting intolerant from tolerant) [19], DEOGEN2 [20], EIGEN [21], MutationAssessor [22], MutPred [23, 24], DANN [25], REVEL (Rare Exome Variant Ensemble Learner) [26], MetaLR [22], MetaSVM [22], MetaRNN [27], and BayesDel [28]. Six meta-analyses or “ensemble” tools were used, including MetaSVM (radial kernel support vector machine) and MetaLR (logistic regression), which integrate 10 component scores and the maximum frequency observed in the 1000 Genomes populations and MetaRNN, which integrates 16 component scores, 8 conservation scores, and allele frequency from three databases. REVEL and BayesDel integrate scores from 13 and nine predictive tools, respectively, and EIGEN integrates protein function scores, evolutionary conservation scores, and allele frequency.

Of the remaining tools, CADD (combined annotation-dependent depletion) [29] and DEOGEN2 [20] can be considered composite scores which integrate several independent scoring systems with diverse features; some examples include functional DNA annotations such as histone modifications and transcription factor binding, as well as protein features reflecting folding, biophysical and structural properties, protein interactions, and pathway information. DANN is an alternative to CADD which utilises deep neural networks as opposed to CADD's SVM (support vector machine) methodology. MutationAssessor predicts pathogenicity based on the evolutionary conservation in protein subfamilies, while GERP++ (genomic evolutionary rate profiling) identifies evolutionarily constrained loci in multiple sequence alignments [30, 31]. GERP++ scores were obtained from the corresponding tracks on the UCSC genome browser [32], and CADD v1.7 [33] scores were obtained from the dedicated web portal (see Web Resources). Lastly, MutPred models protein sequence with SIFT and 14 different structural and functional properties, while VEST4 (variant effect scoring tool) [34] is a machine learning method trained on disease mutations and quantitative features from the SNVBox database including amino acid sequence, conservation, predicted structure, and protein functional annotations [34, 35]. VEST4 scores are available via the CRAVAT web server.

### 2.3. MetaDome Tolerance Analysis

We used MetaDome [36] (version 1.0.1) to examine the potential of HOXC13 p.Arg311Trp to be tolerated. MetaDome is based on the concept of protein domain homology in the human genome, having aggregated homologous Pfam protein domains into “meta-domains.” This method leverages on population variation from the Exome Aggregation Consortium (ExAC) and pathogenic mutations from the Human Gene Mutation Database (HGMD) to create genetic tolerance profiles across human protein “meta-domains” at amino acid resolution. The MetaDome genetic tolerance profiles for human genes were previously derived using 12,164,292 genetic variants from Genome Aggregation Database (gnomAD) [37] and 34,076 pathogenic mutations from ClinVar [38]. The *HOXC13* (ENST00000243056.5/NM_017410.2) MetaDome genetic tolerance profile was investigated via its online portal, where its tolerance landscape is represented as a coloured histogram depicting *d*_*N*_/*d*_*S*_ ratios over sliding windows of 21 residues, calculated for 10 residues left and right of each residue. Scores < 0.7 are considered intolerant.

### 2.4. Predicting Impact on Protein Stability

For sequence-based methods (INPS [39], MUpro [37], and I-Mutant2.0 [40]), the UniProt protein fasta sequence (P31276) and HOXC13 p.Arg311Trp variant were used as input. All three are SVM-based tools. For the remaining structure-based methods, the predicted wild-type structure was provided as a PDB file (details in the Three-Dimensional Modelling section) with the corresponding variant position, R61W. Briefly, DynaMut2 [41] models a range of features including protein dynamics, contact potential stores, and interatomic interactions with graph-based signatures, as does mCSM [42]. MAESTROweb [43] uses a multiagent machine-learning system with features including hydrophilicity, isoelectric point, and statistical energy function. It was run in the “evaluate specific mutations mode.” DUET [44] was run in the default mode which combines SDM [21, 28] (Site-Directed Mutator) and mCSM predictions, as well as in the SDM stand-alone mode. NeEMO (network enthalpic modelling) [45], which uses residue interaction networks, was run with pH = 7.0 and temperature = 20. DDGun3D [46, 47] is an untrained method based on features derived from evolutionary information, ERIS [48] utilises unbiased force field, side-chain packing, and backbone relaxation algorithms, and DeepDDG [49] is a neural network-based method which is trained on more than 5700 manually curated experimental data points. ELASPIC [50] estimates the ΔΔ*G* using more than 70 molecular and energetic features with a stochastic gradient boosting of decision trees algorithm, incorporating protein domain homology and domain-domain interactions, and STRUM [51] utilises a gradient boosting regression approach.

### 2.5. Three-Dimensional Modelling

The three-dimensional structure of human HOXC13 was downloaded from AlphaFold DB [52]. The structures of HOXC13 wildtype (WT) and p.Arg311Trp variant were compared using I-TASSER provided by Yang et al.'s lab [53]. The following standard analysis and modification were performed in PyMOL software (The PyMOL Molecular Graphics System, Version 2.0 Schrödinger, LLC).

### 2.6. Plasmid Construction—*HOXC13* Expression Vectors

*HOXC13* cDNA was isolated by reverse transcription-PCR from total RNA extracted from Human embryonic kidney (HEK) 293T cells with the forward primer containing a KpnI cutting site. The PCR fragment was subsequently cloned into pGEM-T Easy Vector (Promega) following the manufacturer's instructions. After confirming the correct *HOXC13* cDNA by Sanger sequencing, the KpnI/NotI fragment containing *HOXC13* cDNA was then inserted into an expression vector in which *HOXC13* expression is under the control of the CAG promoter. The variants of *HOXC13* cDNA were generated by site-directed mutagenesis using Q5® Site-Directed Mutagenesis Kit (NEB) following the manufacturer's protocol (primers listed in Table S1). After validation by Sanger sequencing, the *HOXC13* cDNA variants were subcloned into the same expression vector as wildtype *HOXC13*.

### 2.7. Plasmid Construction—KRT35-Luciferase Reporter Vector

The promoter region of the *KRT35* gene was amplified by PCR with genomic DNA extracted from HEK 293T cells using PureLink™ Genomic DNA Mini Kit (Thermo Fisher) before being cloned into the pGEM-T Easy Vector (Promega). The KpnI/Bglll fragment containing the *KRT35* promoter was then ligated into the pGL4.10 vector (Promega) to generate the *KRT35*-Luc vector. The pEGFP-C1 vector (Clotech) and the Renilla luciferase-expressing vector pRL-TK (Promega) were used as internal controls in all luciferase assays.

### 2.8. Cell Culture and Transfection

HEK cells 293T purchased from ACTT were cultured in StableCell™ DMEM—high glucose containing 10% fetal bovine serum and 1% penicillin/streptomycin mix (all from Merck) in a humidified incubator at 37°C with 5% CO_2_. Plasmid transfections were performed using the calcium phosphate transfection kit (Merck) according to the manufacturer's instructions, and cells were collected 48 h posttransfection for further analysis. To inhibit proteasome activity, cells were treated with 30 *μ*M MG132 (Merk) for 6 h before being collected for analysis.

### 2.9. Luciferase Assay

The HEK293T cells were cotransfected with the firefly luciferase reporter construct (*KRT35*-Luc) and Renilla luciferase control reporter construct (pRL-TK) together with one of the *HOXC13* expression vectors (*HOXC13*-WT or *HOXC13*-pArg311Trp) or the GFP-control vector (pEGFP-C1). Each group was tested in three independent transfections. Cells were collected 48 h after transfection and seeded into a 96-well plate in triplet for each transfected cell group. The firefly and Renilla luciferase activities were measured in a PHERAstar microplate reader (BMG Labtech) using the Dual-Luciferase Reporter Assay System (Promega) following the manufacturer's instructions.

### 2.10. Quantitative Reverse Transcription PCR (qRT-PCR)

Total RNA was extracted from cells using TRI reagent (Merck), and cDNA was synthesized by ProtoScript® II Reverse Transcriptase (NEB). The qRT-PCR was conducted in StepOnePlus™ Real-Time PCR System (Thermo Fisher) using KiCqStart® SYBR® Green qPCR ReadyMix (Merk). For every experiment, RNA was extracted from three independent transfections, and qRT-PCR was performed in triplet. Data was analysed by normalizing to the housekeeping gene *β*-actin and shown as fold-change over control.

### 2.11. Immunoblotting

Cells were lysed in radioimmunoprecipitation assay (RIPA) buffer (50 mM Tris-HCl, pH 8.0, 150 mM NaCl, 1% Nonidet-P40, 0.5% sodium deoxycholate, and 0.5% SDS) containing protease inhibitor cocktail and 0.2 mM phenylmethanesulfonylfluoride (PMSF) (all from Sigma) on ice for 30 min. Ten micrograms of protein lysate were resolved in 8% SDS-polyacrylamide gel and transferred to polyvinylidene fluoride membrane. The membrane was probed with primary antibody at 4°C overnight after incubation for 1 h in PBS containing 5% milk and 0.1% Tween-20 at room temperature. The secondary antibody was applied for 1 h following the removal of the primary antibody, and finally, the signals were revealed by chemiluminescent detection. Antibodies used are as follows: mouse anti-*α*-Tubulin (Cell Signalling Technology, DM1A, 1 : 3000), rabbit anti-HOXC13 (Life Technologies, PA5103894, 1 : 2000), rabbit anti-*β*-catenin (Cell Signaling, D10A8, 1:1000), HRP-conjugated goat anti-mouse (Santa Cruz Biotechnology, sc-2005, 1:20000), and HRP-conjugated goat anti-rabbit (Santa Cruz Biotechnology, sc-2004, 1:20000).

### 2.12. Statistical Analysis

Statistical analyses were performed by one-way ANOVA using GraphPad Prism 8 with at least three independent biological samples. *P* < 0.05 were considered statistically significant.

## 3. Results and Discussion

### 3.1. Identification of a Rare Homozygous *HOXC13* Variant in a Case of PHNED

**Figure 1:**
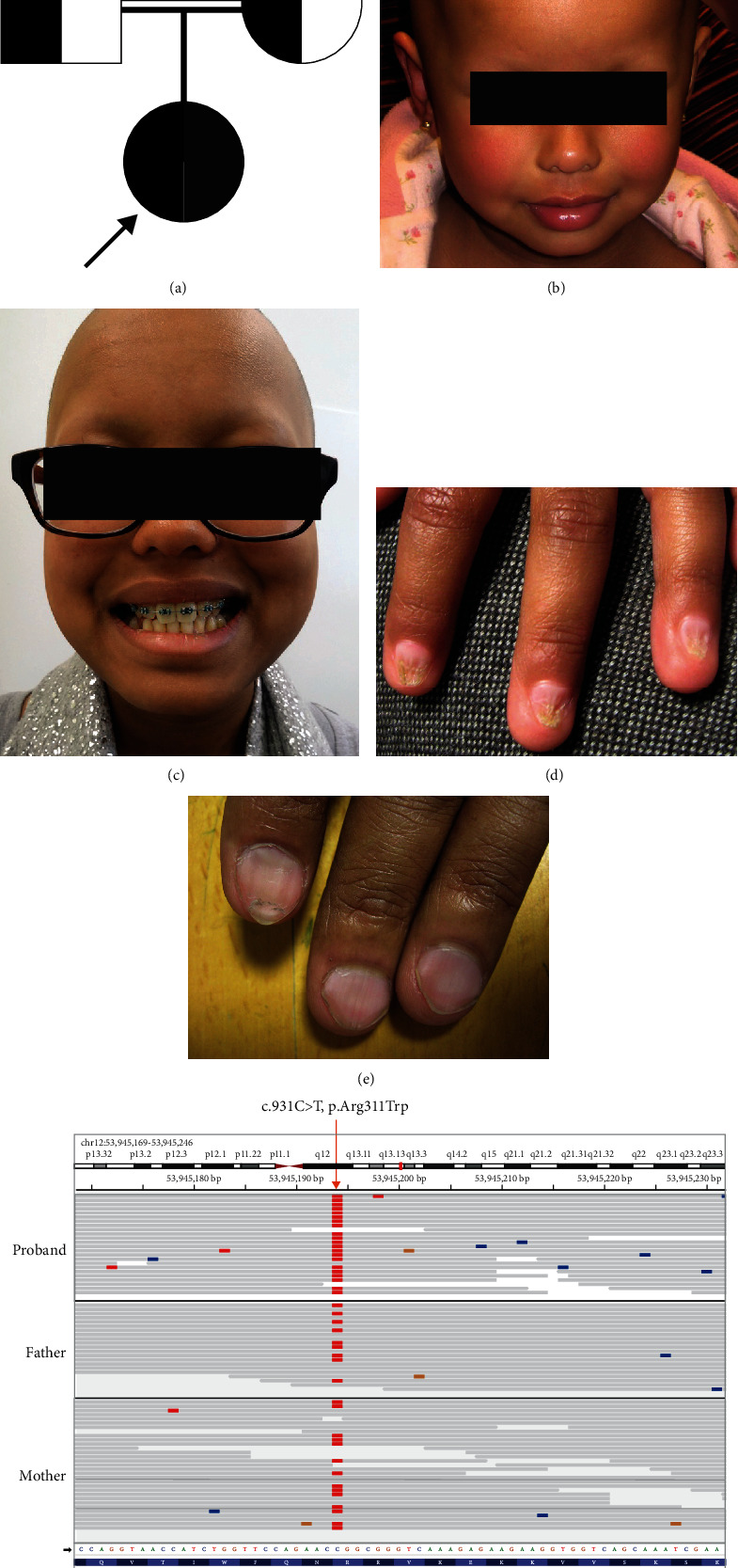
Pedigree and phenotype of proband. (a) The proband is the only child of consanguineous Tamil parents. She carries a homozygous *HOXC13* variant c.931C>T, p.Arg311Trp, while her parents are heterozygotes. (b) The proband at age 27 months, with atrichia. (c) The proband at age 13 years, with atrichia and normal dentition. (d) The proband's nails were dystrophic. (e) The proband's father had isolated koilonychia. (f) An Integrative Genomics Viewer (IGV) screenshot of WGS data from the proband and parents, showing the homozygous genotype of the proband and heterozygous genotype of both parents. The alternative allele (T) is shown in red.

The female proband was the only child of related Sri Lankan parents and was the only affected family member (Figure 1(a)). At birth, the proband was noted to have absent scalp hair, eyelashes, and eyebrows, and all her nails were small and dystrophic (Figures 1(b), 1(c), and 1(d)). She subsequently had some stubble-like hair growth on her scalp, which came away easily when washed. She had no other body hair apart from some secondary sexual hair. Her dentition and sweating have always been normal. There were no other significant features, and she has otherwise had normal growth and development. There was an apparent autosomal dominant paternal family history of koilonychia. Whilst *HOXC13* carriers are reported as having normal hair and nails, the father had koilonychia (Figure 1(e)) but normal hair growth, whereas the mother had normal hair and nails.

Initial genetic investigations showed that the proband had a normal female karyotype, and no pathogenic variants were detected in the ectodermal dysplasia genes *EDA*, *EDAR*, and *GJB6*. Then, trio whole genome sequencing was undertaken within the 100,000 Genomes Project, and a homozygous variant of uncertain clinical significance in *HOXC13* (c.931C>T, p.Arg311Trp) was identified (Figure 1(f)). Both parents were confirmed as heterozygous carriers. Inspection of the gnomAD [54] and the UK Biobank Allele Frequency Browser revealed that the variant c.931C>T, p.Arg311Trp (rs746424242) is extremely rare, being only detected once in heterozygosity in gnomAD (MAF = 0.000006571) and not detected in the UK Biobank Allele Frequency Browser. The variant was predicted to be damaging by *in silico* analysis; however, in the absence of supporting functional data, it was classified as a variant of uncertain significance (VUS).

### 3.2. The *HOXC13* c.931C>T, p.Arg311Trp Variant Is Predicted to Be Pathogenic

**Figure 2:**
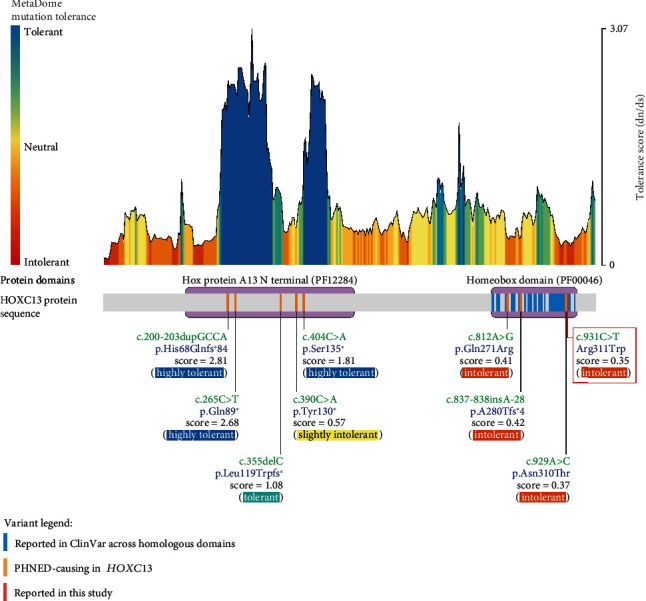
*HOXC13* variant tolerance analysis with MetaDome. MetaDome [36] predicts the Arg311 residue (red vertical line) to be intolerant to sequence variation with a d_N_/d_S_ ratio of 0.35 (scores < 0.7 are considered intolerant). The tolerance landscape (coloured histogram) depicts *d*_*N*_/*d*_*S*_ ratios over sliding windows of 21 residues (see the Material and Methods section). ClinVar [38] variants affecting the protein “meta-domains” found in *HOXC13* (blue vertical lines) are concentrated in the homeobox domain. Orange vertical lines indicate the positions of PHNED *HOXC13* variants. Variants in the Hox protein A13 N terminal domain result in either frameshift or a premature stop codon and affect residues tolerant to variation, while variants in the homeobox domain, where p.Arg311Trp resides, affect residues intolerant to variation.

In order to explore the effects of this variant on *HOXC13* function, we performed a series of computational and experimental studies *in vitro*. Firstly, we queried an extended series of *in silico* variant pathogenicity predictors, including the recently developed deep learning model AlphaMissense [17], to ascertain the potential pathogenicity of the c.931C>T, p.Arg311Trp variant, which consistently predicted the variant to be deleterious (Table 1, see the Material and Methods section for details). We then examined the potential functional impact of the variant on the *HOXC13* gene through MetaDome [36] analysis, which predicted that changes in amino acid Arg311 are not tolerated (*d*_*N*_/*d*_*S*_ ratio = 0.35; *HOXC13*  *d*_*N*_/*d*_*S*_ range = 0.09–3.07) (red box in Figure 2). Moreover, the variant appears within a homeobox “meta-domain” that is highly enriched for pathogenic variants in homologous human protein domains, consistent with this variant being pathogenic (Figure 2, Tables S2 and S3).

This notion is supported by a combined analysis of the *HOXC13* variants previously associated with PHNED [2, 4–6, 55–57], which revealed that all pathogenic variants in regions upstream from the homeobox protein domain result in ablation of the homeobox domain, being either nonsense (p.Gln89∗, p.Tyr130∗, and p.Ser135∗) or frameshift variants (p.His68Glnfs∗84 and p.Leu119Trpfs∗) (Figure 2). A 27.6 kb homozygous deletion spanning the promoter and first exon of *HOXC13* has also been reported as causal of PHNED [5] (not shown). In contrast, the variants in the HOXC13 homeobox domain, where the variant c.931C>T, p.Arg311Trp resides, are mainly missense (c.812A>G, p.Gln271Arg and c.929A>C, p.Asn310Thr) except for one frameshift variant (c.837-838insA28, p.A280Thrfs∗4). These observations support the importance of the HOXC13 homeobox domain in hair and nail development and highlight it as a mutational hotspot where a missense variant can lead to PHNED type 9.

### 3.3. The c.931C>T, p.Arg311Trp Variant Impedes HOXC13 Protein Stability, Reducing Its Transcriptional Activity

**Figure 3:**
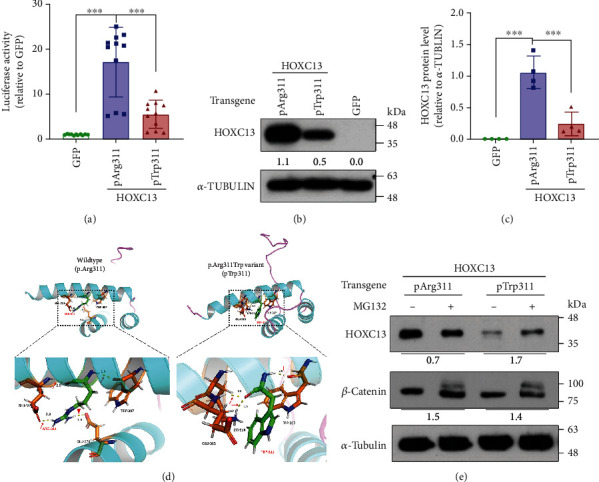
HOXC13 p.Arg311Trp variant altered HOXC13 transcriptional activity and protein stability. (a) Luciferase reporter assay to compare the transcriptional activity between wildtype (WT) and p.Arg311Trp variant of HOXC13 on the *KRT35* promoter. Data are presented as mean ± SD from 9 independent transfection experiments. (b) Immunoblot showing the expression of WT and p.Arg311Trp variant of HOXC13 in transfected HEK293T cells. Numbers below represent expression levels normalized to *α*-TUBULIN. (c) Quantitative analysis on indicated HOXC13 transgenic protein expression. Data are presented as mean ± SD from 4 independent transfection experiments. (d) Predicted homeodomain structures of HOXC13 in wildtype p.Arg311 and variant p.Trp311 (both in red). Arrows: intrahelical interactions; arrowhead: interhelical interaction. (e) Immunoblot showing the expression of indicated transgenic HOXC13 in the presence or absence of proteosome inhibitor MG132 for 6 h. Numbers below represent the ratio of +MG132/-MG132 after normalized to *α*-TUBULIN. *β*-Catenin is used as a positive control for MG132 treatment. ^∗∗∗^*P* < 0.0005 by one-way ANOVA in (a) and (c).

Next, we aimed to obtain direct evidence that the c.931C>T, p.Arg311Trp variant hinders HOXC13 function by comparing the transcriptional activity of the variant to that of WT control. To achieve this, we used luciferase reporter assays in which luciferase expression is driven by the *KRT35* promoter, a direct target of HOXC13 (Figure S1A) [8]. This plasmid was cotransfected with an expression vector containing either the WT or the c.931C>T, p.Arg311Trp variant *HOXC13* into HEK293T cells. Given that the *KRT35* promoter is directly activated by HOXC13 [8], this experimental design allows the comparison of the transcriptional activity of WT and variant HOXC13 by measuring luciferase expression. These experiments showed that the c.931C>T, p.Arg311Trp variant significantly reduced luciferase reporter activity (Figure 3(a)), demonstrating that the c.931C>T, p.Arg311Trp variant indeed impairs HOXC13 function as a transcriptional activator. To explore the underlying mechanisms, we also compared the expression levels of the WT and c.931C>T, p.Arg311Trp variant *HOXC13* transgenes in the transfected HEK293 cells. Surprisingly, the c.931C>T, p.Arg311Trp variant protein was detected at a much lower level than the WT control, whereas its corresponding mRNA showed higher expression than the WT mRNA. This excludes technical artefacts and the possibility of reduced transcriptional rate or stability as causal mechanisms for the observed differences in protein levels (Figure 3(b), Figure S1B). In addition, this difference in protein expression was consistent and reproducible in multiple transfection experiments (Figure 3(c)). Furthermore, when inhibiting the proteasome with MG132, the level of c.931C>T, p.Arg311Trp variant protein was clearly increased, in contrast with the WT protein (Figure 3(d)). Altogether, these results indicate that the c.931C>T, p.Arg311Trp variant may impede HOXC13 protein stability.

To further investigate this, we compared the HOXC13 homeobox domain structures of WT and the c.931C>T, p.Arg311Trp variant using protein modelling. In WT protein, there is an interhelical connection between Arg311 in *α*-helix 3 and Glu276 in *α*-helix 1, which is lost in the c.931C>T, p.Arg311Trp variant (Figure 3(e)). Predicting protein stability *in silico* is challenging [58], therefore we used an assortment of web-based tools to obtain the consensus prediction (see the Material and Methods section). A total of 9 out of 13 *in silico* tools predicted the *HOXC13* c.931C>T, p.Arg311Trp variant to be destabilising based on a threshold of *ΔΔG*< -0.5 (see ref [59] for a discussion of thresholds) (Table 2).

Jointly, the *in silico* analyses along with *in vitro* experimental results suggest that the c.931C>T, p.Arg311Trp variant may produce an unstable HOXC13 protein. In support of this, inhibition of proteasome activity increased the protein level of this variant but not the WT control (Figure 3(e)). Interestingly, inhibiting proteasome activity did not show a clear effect on the previously reported *HOXC13* c. 929A>C, p.Asn310Thr variant, which affects an amino acid residue adjacent to the Arg311, although both of these variants reduced HOXC13 transcriptional activation on *KRT35* (Figure S1C–1F). This observation is consistent with previous reports showing that overexpression of the *HOXC13* c. 929A>C, p.Asn310Thr variant is not associated with changes in protein stability [60].

Various molecular mechanisms have been described for different PHNED-associated variants in the *HOXC13* gene, including loss of protein via nonsense-mediated mRNA decay [5], impaired nuclear translocation [2], and altered DNA-binding [4]. For example, nonsense-mediated decay was suggested to account for the dramatic reduction in *HOXC13* mRNA in skin tissue and the near absence of protein staining in the hair follicles of a patient with a *HOXC13* nonsense variant [5]. In another patient with a *HOXC13* frameshift mutation, studies showed that expressing the mutant HOXC13 in cultured cells results in the accumulation of the protein in the cytoplasm, failing to translocate into the nucleus to upregulate its target genes [2]. Here, our results uncover *HOXC13* c.931C>T, p.Arg311Trp as a novel pathogenic variant that reduces protein stability and, hence, decreases the potential to activate target gene transcription. We note that although both Asn310 and Arg311 reside within the homeobox domain (Figure 2), Arg311 is involved in interhelical interaction, while Asn310 is predicted to be directly involved in “Specific DNA base contacts” (as reported on InterPro, not shown). Furthermore, posttranslational methylation of arginine residues is a common mechanism involved in protein stability through inhibiting proteasomal degradation [61]. These may account for the difference in the mechanisms by which HOXC13 Asn310 and Arg311 residues affect HOXC13 function and protein stability.

## 4. Conclusion

The evidence to date shows that pathogenic *HOXC13* variants can affect the transcription factor's activity through several different molecular mechanisms. This study demonstrates that the homozygous variant *HOXC13* c.931C>T, p.Arg311Trp is causative of PHNED, through a new molecular mechanism of impaired protein stability.

## 5. Web Resources

AlphaMissense, https://zenodo.org/records/8360242

CADD v1.7, https://cadd.bihealth.org/snv

CRAVAT, http://cravat.us/CRAVAT/

DDGun3D, https://folding.biofold.org/ddgun/

DeepDGG, http://protein.org.cn/ddg.html

DUET, https://biosig.lab.uq.edu.au/duet

DynaMut2, https://biosig.lab.uq.edu.au/dynamut2/

ELASPIC, http://elaspic.kimlab.org/

ERIS, https://dokhlab.med.psu.edu/eris/login.php

Ensembl Variation – Pathogenicity predictions, http://www.ensembl.org/info/genome/variation/prediction/protein_function.html

Exome Aggregation Consortium (ExAC), https://exac.broadinstitute.org/

GnomAD, https://gnomad.broadinstitute.org/

HGMD, Human Genome Mutation Atlas http://www.hgmd.cf.ac.uk/ac/index.php

I-Mutant2.0, http://gpcr.biocomp.unibo.it/cgi/predictors/I-Mutant2.0/I-Mutant2.0.cgi

INPS, https://inpsmd.biocomp.unibo.it/inpsSuite

MAESTROweb, https://pbwww.services.came.sbg.ac.at/maestro/web/maestro

MetaDome, https://stuart.radboudumc.nl/metadome

mCSM, https://biosig.lab.uq.edu.au/mcsm/

MUpro, https://www.ics.uci.edu/%7Ebaldig/mutation.html

NeEMO, http://protein.bio.unipd.it/neemo/

OMIM, https://omim.org/

PolyPhen-2, http://genetics.bwh.harvard.edu/pph2/

STRUM, https://zhanggroup.org/STRUM/

UCSC Genome Browser, https://genome.ucsc.edu/

UniProt, https://www.uniprot.org/

Variant Effect Predictor (VEP) from Ensembl, https://www.ensembl.org/

VarSome, https://varsome.com/

UK Biobank Allele Frequency Browser, https://afb.ukbiobank.ac.uk/

## Figures and Tables

**Table 1:** Predicted pathogenicity of the HOXC13 c.931C>T, p.Arg311Trp variant.

**Software**	**Range (threshold)**	**Prediction**
Meta-analysis tools		
MetaSVM	−2.0–3.0 (> 0.0)	1.05
MetaLR	0.0–1.0 (> 0.5)	0.98
metarnn	0.0–1.0 (> 0.5)	0.95
REVEL	0.0–1.0 (> 0.5)	0.87
BayesDel	−1.12–0.75 (> 0.07)	0.48
Eigen	0.0–1.0 (> 0.5)	0.94
Individual predictors		
AlphaMissense	0.0–1.0 (> 0.564)	0.9993—pathogenic
SIFT	0.0–1.0 (< 0.05)	0.0—deleterious
PolyPhen-2	0.0–1.0 (> 0.45)	1.0—probably damaging
VEST4	0.0–1.0 (< 0.05)	0.003
Cadd v1.7	1–99 (> 30)	34
DANN	0.0–1.0 (> 0.5)	0.9992
gerp++	−12.3–6.17 (> 2)	5.1
MutationAssessor	0.0–1.0 (> 0.65)	0.99
Mutpred	0.0–1.0 (> 0.5)	0.803
DEOGEN2	0.0–1.0 (> 0.5)	0.945

*Note:* the table shows the output (PREDICTION) of each tool as a score, ranked score, or *p* value (VEST4), along with the range and threshold for defining the variant as likely pathogenic, according to guidelines reported by the authors or suggested by the Ensembl guide to predicting protein function (link in the Web Resources section).

**Table 2:** Predicted impact of the HOXC13 p.Arg311Trp amino acid change on protein stability.

**Software**	ΔΔ**G(kcal/mol)**^a^
Sequence based	
INPS	−0.70
MUpro	−1.05
I-Mutant2.0	−0.29
Structure-based	
DUET	−0.60
SDM (via DUET)	0.45
mCSM	−0.77
NeemO	−1.62
MAESTROweb	−1.01^b^
DynaMut2	−0.64
DDGun3d	−0.30
Eris	−6.96
STRUM	−0.47
ELASPIC	−0.71

^a^ΔΔ*G* = Δ*G*_variant_–Δ*G*_wild−type_ with Δ*G* representing the change in Gibbs free energy (kcal/mol) between the folded and unfolded states. Note that −0.5 < ΔΔ*G* < 0.5 are not considered to be significant, and ΔΔ*G* < −0.5 is considered destabilising.

^b^MAESTROweb provides a *C*_pred_ confidence score of 0.86, which ranges from 0.0 to 1.0—highly reliable.

## Data Availability

Whole-genome sequence data supporting the findings of this study are available within the Genomics England Research Environment, a secure workspace, to members of the Genomics England Research Network (academics/healthcare professionals) or the Discovery Forum (industry). Researchers can apply via the Genomics England website: https://www.genomicsengland.co.uk/research.

## Supporting Information

Supporting Information Additional supporting information can be found online in the Supporting Information section. Additional supporting information can be found online in the Supporting Information section.
