# Supplementary material for: Homozygous HOXC13 Variant Causes Pure Hair and Nail Ectodermal Dysplasia via Reduction in Protein Stability
Source: Hum Mutat. 2024 Jul 1;2024:6420246. doi: 10.1155/2024/6420246 (PMC11919099; doi:10.1155/2024/6420246)
Supplement: Supporting Information — Additional supporting information can be found online in the Supporting Information section. Additional supporting information can be found online in the Supporting Information section. [file 6420246.f1.zip › Supplementary Materials-revised/Table S2.pdf]

Table S2. Pathogenic ClinVar SNVs at homologous positions from MetaDome analysis.

Gene	Position	Variant	Residue change	Type	ClinVar ID
HNF1A	chr12:121432064	C>T	Arg>Trp	missense	265193
NKX2-5	chr5:172659981	G>C	Arg>Pro	missense	190834
SHOX	chrX:601571	C>T	Arg>Trp	missense	9879
OTX2	chr14:57269058	C>G	Arg>Gly	missense	9516
PAX6	chr11:31815335	C>T	Arg>*	nonsense	279862
CRX	chr19:48342592	C>T	Arg>Trp	missense	7422
NOBOX	chr7:144096940	G>A	Arg>His	missense	1083
HNF1A	chr12:121432065	G>A	Arg>Gln	missense	449403
SIX3	chr2:45170013	G>C	Arg>Pro	missense	6094
ALX4	chr11:44289157	C>T	Arg>*	nonsense	5020
OTX2	chr14:57269058	C>T	Arg>*	nonsense	190250
