# Supplementary material for: Homozygous HOXC13 Variant Causes Pure Hair and Nail Ectodermal Dysplasia via Reduction in Protein Stability
Source: Hum Mutat. 2024 Jul 1;2024:6420246. doi: 10.1155/2024/6420246 (PMC11919099; doi:10.1155/2024/6420246)
Supplement: Supporting Information — Additional supporting information can be found online in the Supporting Information section. Additional supporting information can be found online in the Supporting Information section. [file 6420246.f1.zip › Supplementary Materials-revised/Table S3.pdf]

Table S3. gnomAD SNVs at homologous positions from MetaDome analysis.

Gene	Position	Variant	Residue change	Type	gnomAD Allele Frequency
NKX2-1	chr14:36986965	G>A	His>Tyr	missense	0.000004
VAX2	chr2:71159919	G>C	Arg>Pro	missense	0.000004
NOBOX	chr7:144096940	C>G	Arg>Pro	missense	0.000004
NKX2-4	chr20:21376896	G>A	His>Tyr	missense	0.000096
NKX2-8	chr14:37050422	A>G	His>His	synonymous	0.000008
HOXA2	chr7:27140894	C>G	Arg>Arg	synonymous	0.000004
GBX1	chr7:150845832	T>C	Arg>Arg	synonymous	0.000041
NOBOX	chr7:144096940	C>T	Arg>His	missense	0.000163
PDX1	chr13:28498577	C>T	Arg>Arg	synonymous	0.000004
CERS3	chr15:101024803	C>T	Arg>Gln	missense	0.000008
HOXB2	chr17:46620920	C>G	Arg>Pro	missense	0.000004
ZHX1	chr8:124267182	T>C	Gln>Gln	synonymous	0.000004
OTX1	chr2:63282652	G>A	Arg>His	missense	0.000004
GBX1	chr7:150845833	C>T	Arg>Gln	missense	0.000024
ZHX1	chr8:124266332	T>C	Lys>Glu	missense	0.000004
ALX3	chr1:110604169	C>T	Arg>His	missense	0.000024
CDX1	chr5:149563059	G>A	Arg>Gln	missense	0.000004
HOXC10	chr12:54383156	C>T	Arg>Cys	missense	0.000004
VAX2	chr2:71159918	C>T	Arg>Cys	missense	0.000035
GSC2	chr22:19136593	G>A	Arg>Cys	missense	0.000015
ZFHX3	chr16:72829993	A>T	Thr>Thr	synonymous	0.000073
EN1	chr2:119600631	C>T	Lys>Lys	synonymous	0.00002
SIX3	chr2:45170014	G>C	Arg>Arg	synonymous	0.000004
LEUTX	chr19:40276353	C>T	Gln>*	nonsense	0.000007
LHX2	chr9:126794714	G>A	Ala>Thr	missense	0.000004
POU1F1	chr3:87309127	G>A	Arg>Trp	missense	0.000008
SHOX	chrX:601573	G>A	Arg>Arg	synonymous	0.000004
DBX1	chr11:20178098	G>A	Arg>*	nonsense	0.000004
CRX	chr19:48342593	G>A	Arg>Gln	missense	0.000004
CERS4	chr19:8320561	G>T	Arg>Leu	missense	0.000004
ARGFX	chr3:121304885	G>A	Arg>Gln	missense	0.000037
BSX	chr11:122848577	C>T	Arg>Gln	missense	0.000004
CERS4	chr19:8320560	C>T	Arg>Cys	missense	0.000098
DUXA	chr19:57666724	C>T	Arg>Gln	missense	0.000004
POU2F3	chr11:120180221	C>A	Arg>Arg	synonymous	0.000016

POU6F1	chr12:51584083	G>T	Arg>Arg	synonymous	0.000004
HMX3	chr10:124897007	C>T	Arg>Arg	synonymous	0.000004
SHOX2	chr3:157818084	C>A	Arg>Leu	missense	0.000004
HOXB13	chr17:46804208	G>A	Arg>Cys	missense	0.000008
ARGFX	chr3:121304884	C>T	Arg>Trp	missense	0.000009
HOPX	chr4:57514947	C>A	Arg>Leu	missense	0.000004
ZHX1	chr8:124266330	C>T	Lys>Lys	synonymous	0.000008
HNF1A	chr12:121432065	G>A	Arg>Gln	missense	0.000008
HOXB7	chr17:46685296	G>A	Arg>Trp	missense	0.000008
ZFHX4	chr8:77765696	C>T	Thr>Met	missense	0.000004
BARX1	chr9:96715034	G>A	Arg>Trp	missense	0.000004
NKX2-8	chr14:37050423	T>C	His>Arg	missense	0.000013
SHOX2	chr3:157818084	C>T	Arg>Gln	missense	0.000004
HOXA4	chr7:27169009	C>T	Arg>Arg	synonymous	0.00002
PAX3	chr2:223086091	G>A	Arg>Cys	missense	0.000004
PAX4	chr7:127253130	T>G	Arg>Arg	synonymous	0.000004
HOXA5	chr7:27181529	C>T	Arg>Arg	synonymous	0.000004
PBX2	chr6:32155149	C>T	Lys>Lys	synonymous	0.000008
ISX	chr22:35480391	C>T	Gln>*	nonsense	0.000004
POU2F3	chr11:120180221	C>T	Arg>*	nonsense	0.000008
HOXA10	chr7:27211591	C>T	Arg>His	missense	0.000004
ISX	chr22:35480392	A>C	Gln>Pro	missense	0.000004
NANOGNB	chr12:7923058	G>A	Thr>Thr	synonymous	0.000081
ZFHX3	chr16:72829995	T>C	Thr>Ala	missense	0.000004
ZFHX4	chr8:77765697	G>T	Thr>Thr	synonymous	0.000045
CERS3	chr15:101024804	G>A	Arg>Trp	missense	0.000041
HOXA9	chr7:27203272	G>T	Arg>Ser	missense	0.000004
HOXC12	chr12:54350294	C>T	Arg>Trp	missense	0.00002
VENTX	chr10:135053457	C>T	Arg>Cys	missense	0.000029
LHX8	chr1:75622594	G>T	Cys>Phe	missense	0.000004
PITX2	chr4:111539827	A>G	Arg>Arg	synonymous	0.000012
CERS4	chr19:8320561	G>A	Arg>His	missense	0.000033
ZFHX4	chr8:77767125	A>T	Thr>Thr	synonymous	0.000016
VTN	chr17:26691655	G>A	Arg>Cys	missense	0.000016
GSC	chr14:95234969	G>C	Arg>Arg	synonymous	0.000004
HOXB1	chr17:46607055	G>A	Arg>*	nonsense	0.000004
EVX1	chr7:27285520	C>G	Arg>Gly	missense	0.000004
PITX1	chr5:134364996	G>T	Arg>Arg	synonymous	0.122703
HOXB13	chr17:46804206	G>A	Arg>Arg	synonymous	0.000004
VENTX	chr10:135053458	G>A	Arg>His	missense	0.000004

DLX3	chr17:48069206	C>T	Arg>His	missense	0.000041
ALX1	chr12:85680646	C>A	Arg>Arg	synonymous	0.000004
ZHX3	chr20:39831570	T>C	Arg>Gly	missense	0.000004
HESX1	chr3:57232308	G>A	Arg>Trp	missense	0.00002
HOXD3	chr2:177036436	C>G	Arg>Gly	missense	0.000004
MEOX1	chr17:41719379	G>T	Arg>Arg	synonymous	0.000008
CERS5	chr12:50536904	C>T	Arg>Arg	synonymous	0.000004
VAX2	chr2:71159919	G>A	Arg>His	missense	0.000147
HOXB9	chr17:46700309	G>A	Arg>Trp	missense	0.000016
NANOGNB	chr12:7923057	C>T	Thr>Met	missense	0.000175
ALX3	chr1:110604170	G>A	Arg>Cys	missense	0.000004
ZHX1	chr8:124266332	T>G	Lys>Gln	missense	0.000004
NKX2-4	chr20:21376894	G>A	His>His	synonymous	0.000507
HOXA4	chr7:27169011	G>A	Arg>Trp	missense	0.000004
LHX4	chr1:180240987	A>C	Arg>Ser	missense	0.000004
PRRX2	chr9:132482891	G>A	Arg>His	missense	0.000004
HOXB7	chr17:46685296	G>T	Arg>Arg	synonymous	0.000004
ZHX3	chr20:39831922	A>G	Arg>Arg	synonymous	0.000008
HOXB3	chr17:46628276	C>G	Arg>Pro	missense	0.000004
HOXB9	chr17:46700308	C>A	Arg>Leu	missense	0.000004
ZFHX4	chr8:77765697	G>A	Thr>Thr	synonymous	0.000004
HOXC11	chr12:54369129	A>C	Arg>Arg	synonymous	0.000008
GBX2	chr2:237074710	T>C	Arg>Arg	synonymous	0.000004
ALX4	chr11:44289156	C>T	Arg>Gln	missense	0.000004
POU6F1	chr12:51584082	C>T	Arg>Gln	missense	0.000008
DUXA	chr19:57670630	C>T	Arg>Gln	missense	0.000037
HOXA7	chr7:27194680	G>C	Arg>Gly	missense	0.000004
BARX2	chr11:129312791	C>T	Arg>Cys	missense	0.000008
VSX2	chr14:74726321	G>A	Arg>His	missense	0.000041
OTX1	chr2:63282653	C>T	Arg>Arg	synonymous	0.000012
HOXC12	chr12:54350295	G>A	Arg>Gln	missense	0.000012
ARGFX	chr3:121304886	G>A	Arg>Arg	synonymous	0.000005
HOXC11	chr12:54369131	A>G	Arg>Arg	synonymous	0.000008
NKX2-2	chr20:21492846	G>A	His>His	synonymous	0.000008
ALX1	chr12:85680646	C>T	Arg>*	nonsense	0.000004
BSX	chr11:122848578	G>A	Arg>Trp	missense	0.000004
HOXB8	chr17:46690705	C>T	Arg>Arg	synonymous	0.000004
PBX2	chr6:32155150	T>C	Lys>Arg	missense	0.000004
HOXB7	chr17:46685294	C>T	Arg>Arg	synonymous	0.000004
HOXB3	chr17:46628277	G>A	Arg>Trp	missense	0.000004

HOXC8	chr12:54405035	G>A	Arg>Gln	missense	0.000004
SIX4	chr14:61189973	G>T	Arg>Ser	missense	0.000008
PROP1	chr5:177420033	G>A	Arg>Cys	missense	0.00007
NKX3-1	chr8:23538914	T>C	Arg>Arg	synonymous	0.000004
HOXD13	chr2:176959405	C>T	Arg>*	nonsense	0.000065
HOXC9	chr12:54396402	C>T	Arg>*	nonsense	0.000004
NKX2-5	chr5:172659982	G>T	Arg>Arg	synonymous	0.000012
HDX	chrX:83599448	T>C	Arg>Arg	synonymous	0.000006
DUXA	chr19:57666724	C>A	Arg>Leu	missense	0.000004
NOBOX	chr7:144096939	G>A	Arg>Arg	synonymous	0.000008
HOXC10	chr12:54383157	G>A	Arg>His	missense	0.000004
HOXD11	chr2:176973802	C>T	Arg>Cys	missense	0.000024
HOXA6	chr7:27185363	G>C	Arg>Gly	missense	0.000008
OTX2	chr14:57269057	C>T	Arg>Gln	missense	0.000041
MEOX1	chr17:41719378	C>T	Arg>Gln	missense	0.000025
SATB1	chr3:18390868	G>A	Gln>*	nonsense	0.000004
POU6F1	chr12:51584083	G>A	Arg>Trp	missense	0.000008
SHOX	chrX:601571	C>T	Arg>Trp	missense	0.000004
ZFHX4	chr8:77765697	G>C	Thr>Thr	synonymous	0.000004
PBX4	chr19:19675877	T>C	Lys>Glu	missense	0.00002
ZHX3	chr20:39831923	C>T	Arg>His	missense	0.000049
HOXD12	chr2:176965434	G>A	Arg>Arg	synonymous	0.000012
VSX2	chr14:74726320	C>T	Arg>Cys	missense	0.000012
HOXD13	chr2:176959407	A>G	Arg>Arg	synonymous	0.000004
VTN	chr17:26691654	C>T	Arg>His	missense	0.000042
HOXA11	chr7:27222483	T>G	Arg>Arg	synonymous	0.000008
HLX	chr1:221057558	C>T	Arg>Trp	missense	0.000004
MEOX2	chr7:15652215	T>G	Arg>Arg	synonymous	0.000012
HOPX	chr4:57514948	G>A	Arg>Cys	missense	0.000033
HOXA9	chr7:27203270	G>A	Arg>Arg	synonymous	0.000008
VENTX	chr10:135053458	G>T	Arg>Leu	missense	0.000008
ZHX3	chr20:39832492	T>G	Gln>His	missense	0.000004
ANHX	chr12:133803678	A>T	Tyr>Asn	missense	0.000008
ZHX1	chr8:124266054	G>T	Thr>Thr	synonymous	0.000004
ZHX1	chr8:124266054	G>C	Thr>Thr	synonymous	0.000008
NOTO	chr2:73437920	C>T	Arg>Cys	missense	0.000013
PBX4	chr19:19675877	T>G	Lys>Gln	missense	0.000004
LHX4	chr1:180240986	G>A	Arg>Lys	missense	0.000004
HDX	chrX:83599449	C>T	Arg>Gln	missense	0.000017
LHX8	chr1:75622595	T>C	Cys>Cys	synonymous	0.000016

MSX1	chr4:4864627	C>T	Arg>Arg	synonymous	0.000004
CERS5	chr12:50536905	C>T	Arg>Gln	missense	0.000012
POU5F2	chr5:93076487	G>A	Arg>Arg	synonymous	0.000004
LBX2	chr2:74725243	C>G	Arg>Arg	synonymous	0.000004
HOXD4	chr2:177017515	C>T	Arg>Trp	missense	0.000016
ZFHX3	chr16:72829702	G>A	Ala>Ala	synonymous	0.000004
HOPX	chr4:57514947	C>T	Arg>His	missense	0.000053
NANOGNB	chr12:7923056	A>C	Thr>Pro	missense	0.000013
ALX1	chr12:85680647	G>A	Arg>Gln	missense	0.000004
HOXB9	chr17:46700308	C>T	Arg>Gln	missense	0.000016
DUXA	chr19:57666725	G>A	Arg>*	nonsense	0.00002
HOXB6	chr17:46673861	G>T	Arg>Arg	synonymous	0.000004
ALX4	chr11:44289156	C>A	Arg>Leu	missense	0.000004
HOXB5	chr17:46669647	C>A	Arg>Leu	missense	0.000004
