# Supplementary material for: Homozygous HOXC13 Variant Causes Pure Hair and Nail Ectodermal Dysplasia via Reduction in Protein Stability
Source: Hum Mutat. 2024 Jul 1;2024:6420246. doi: 10.1155/2024/6420246 (PMC11919099; doi:10.1155/2024/6420246)
Supplement: Supporting Information — Additional supporting information can be found online in the Supporting Information section. Additional supporting information can be found online in the Supporting Information section. [file 6420246.f1.zip › Supplementary Materials-revised/Table S1.pdf]

Table S1. Primers used in this study.

	Forward primer (5’- 3')	Reverse primer (5’- 3')
HOXC13 cDNA isolation		
	AGCTGGTACCATGACGACTTCGCTG	GGTGGTCAGGTGGAGTGGAGATGAG
HOXC13 directed Site-mutagenesis		
p.Arg311Trp	GTTCCAGAACTGGCGGGTCAA	CAGATGGTTACCTGGCGC
p.Asn310Thr	TGGTTCCAGACCCGGCGGGTC	GATGGTTACCTGGCGCTCAG
KRT35 promoter isolation		
	AATGGTACCGACAGTGGCTGAACTCCCAT	GCAGATCTACCCCAAAGCAGAGAAGCAG
RT-qPCR		
HOXC13	GCCACCACGAACCTCTCTGA	TTTGGCAGAGGGAAAAAGATCTCA
β-ACTIN	TGTCTGGCGGCACCACCATG	AGGATGGAGCCGCCGATCCA
