# Supplementary material for: Homozygous HOXC13 Variant Causes Pure Hair and Nail Ectodermal Dysplasia via Reduction in Protein Stability
Source: Hum Mutat. 2024 Jul 1;2024:6420246. doi: 10.1155/2024/6420246 (PMC11919099; doi:10.1155/2024/6420246)
Supplement: Supporting Information — Additional supporting information can be found online in the Supporting Information section. Additional supporting information can be found online in the Supporting Information section. [file 6420246.f1.zip › Supplementary Materials-revised/Figure S1.pdf]

Figure S1

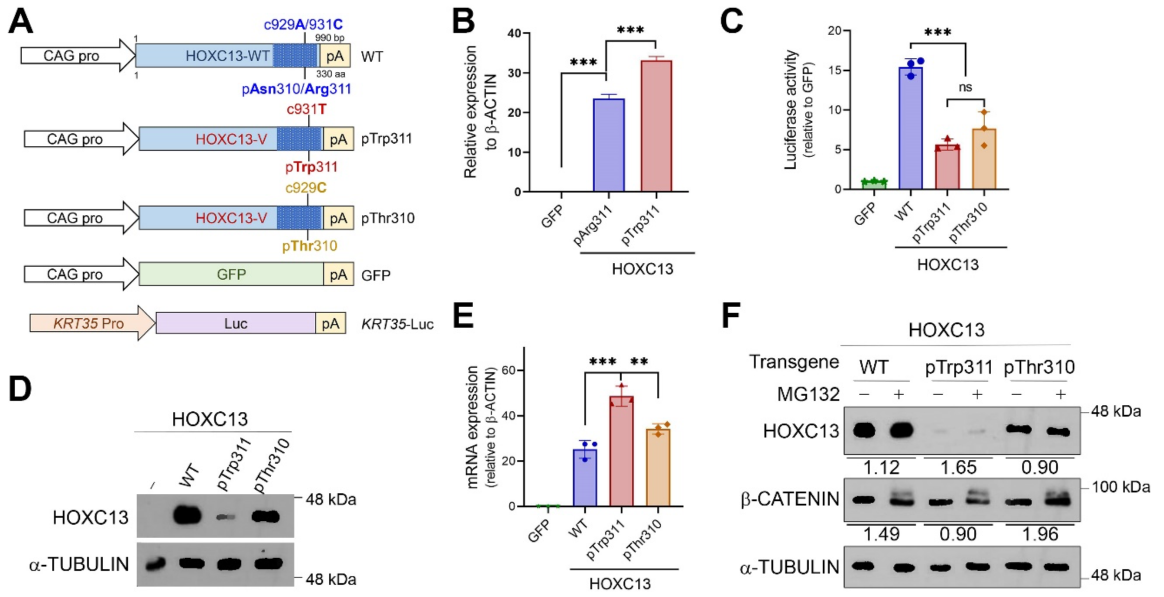

HOXC13 p.Arg311Trp variant reduced protein stability

(A) Schematic depicting the expression constructs used in luciferase reporter assays.

(B) Transgenic mRNA expression of *HOXC13* wildtype (WT) and p.Arg311Trp variant by RT-qPCR, corresponding with the protein expression in Figure 3B from the same transfection. Error bars represent standard derivation from 3 technical PCR replicates.

(C) Luciferase reporter assays with indicated HOXC13 variants on the *KRT35* promoter. Data are presented as mean ± SD from 3 independent transfection experiments.

(D) Immunoblot showing protein expression of indicated HOXC13 variants in transfected HEK293T cells. Numbers below represent expression levels normalized to α-TUBULIN.

(E) RT-qPCR showing *HOXC13* mRNA expression of indicated transgenes. Data are presented as mean ± SD from 3 independent transfection experiments.

(F) Immunoblot showing the expression of indicated transgenic HOXC13 in the presence or absence of proteosome inhibitor MG132 for 6 hours. Data are presented similarly as Figure 3E.
